# The Effect of Mirror Visual Feedback on Spatial Neglect for Patients after Stroke: A Preliminary Randomized Controlled Trial

**DOI:** 10.3390/brainsci13010003

**Published:** 2022-12-20

**Authors:** Kenneth N. K. Fong, K. H. Ting, Xinfei Zhang, Christina S. F. Yau, Leonard S. W. Li

**Affiliations:** 1 Department of Rehabilitation Sciences, The Hong Kong Polytechnic University, Hong Kong SAR, China; 2 University Research Facility in Behavioral and Systems Neuroscience, The Hong Kong Polytechnic University, Hong Kong SAR, China; 3 Guangdong 999 Brain Hospital, Guangzhou 510510, China; 4 Occupational Therapy Department, Tung Wah Hospital, Hospital Authority, Hong Kong SAR, China; 5 Department of Medicine, Tung Wah Hospital, Hospital Authority, Hong Kong SAR, China

**Keywords:** mirror visual feedback, stroke, spatial neglect, allocentric neglect, bilateral arm movement

## Abstract

We investigated the effects of mirror visual feedback (MVF), with reference to using a glass wall or a covered mirror, on the reduction of spatial neglect for patients with stroke. A total of 21 subacute patients with left spatial neglect after right-hemispheric stroke were randomly assigned to 3 groups: MVF, sham 1 (viewing the hemiparetic arm through the transparent glass during bilateral arm movement) and sham 2 (using a covered mirror). The 3-week treatment program for all groups consisted of 12 sessions of movement tasks for the hemiparetic arm graded according to the severity of arm impairments. Blinded assessments were administered at pre/post and a three-week follow-up. The results showed that there was no significant advantage for MVF than sham 1; however, MVF was more beneficial than sham 2, as shown by the line crossing (*p* = 0.022). Improvement in discriminating the left-gap figures on the left and right side of the page in the Gap Detection Test was greater in MVF than using the covered mirror (*p* = 0.013; *p* = 0.010), showing a slight advantage of MVF in alleviating allocentric symptoms. Our study confirms that MVF was superior to using a covered mirror as a method for reducing spatial neglect and in alleviating its allocentric symptoms, but no significant advantage over bilateral arm movement through transparent glass was found. Further research in comparing their therapeutic effects is warranted.

## 1. Introduction

Spatial neglect, or unilateral neglect, is usually seen as a cluster of attention deficits with heterogeneous symptoms that occurs after stroke. The prevalence rate of spatial neglect after unilateral stroke is 30%, 30% after right brain damage and 18% after left brain damage [1]. Spatial neglect is usually defined according to input and output modalities, spatial representation, or range of space [2]. The neglect of input is commonly seen as “sensory”, which includes visual neglect or inattention of the contralesional side, and the output is commonly seen as “motor neglect”. Spatial neglect can also be divided into egocentric and allocentric types, according to a reference framework of spatial representation; or into personal, peripersonal, and extrapersonal neglect, according to the range of space to the body of the viewer [2]. Patients with egocentric (body-centered) neglect will neglect their body or personal space, or the far end of the space opposite to their lesion side, while individuals with allocentric (stimulus-centered) neglect will neglect the contralesional side of a stimulus, either in regard to their peri-personal or extrapersonal space, regardless of the location of stimulus in relation to the body of the viewer [3].

‘Hemispatial’ theory accounts for the neglect phenomenon by proposing right hemisphere dominance for spatial attention: whereas the right hemisphere directs visual attention to both left and right spatial fields, the left hemisphere directs visual attention to the right spatial field only [4]. Recent findings have shown that spatial neglect is more strongly associated with the disruption of inter-hemispheric connectivity in the dorsal attention network [5]. The reason for neglect is best explained by reference to the ‘interhemispheric rivalry model of spatial attention’ according to which each hemisphere directs spatial attention toward the contralateral visual field and is balanced through reciprocal inhibition [6]. The lowered levels of excitability in the ipsilesional side, due to damage and a decrease in input, will cause more excitability to be released in the contralesional side through transcallosal inhibition. This condition results in relatively higher levels of excitability in the contralesional hemisphere and lower levels of excitability in the ipsilesional side, leading to an imbalance in visual spatial attention between the hemispheres, hence, a spatial neglect toward the ipsilesional field [7].

A recent review of 65 trials concluded that there is no strong evidence that a specific treatment for spatial neglect is more efficacious and that the quality of evidence on use of these treatments are found to be of low quality due to the small size of studies and heterogeneous factors including participant characteristics, types of treatments, and assessments used to measure changes [8]. Nevertheless, the findings of our published meta-analysis of rehabilitation interventions for neglect do show that bottom-up interventions that aim to increase spatial attention of the body space and promote awareness of the surrounding area of the neglect field could lead to more balanced interhemispheric symmetry of excitability [9].

Mirror therapy has been proved to be a useful and inexpensive intervention for upper limb hemiparesis following stroke [10,11,12]. It has been classified as a kind of body awareness intervention for spatial neglect that focused on proprioception and awareness of the body in space or in relation to midline [8]. Recently, a systematic review of the effectiveness of mirror therapy in the treatment of spatial neglect after stroke has shown that limited trials have been published [13]. Hence, little can be understood from the literature about the actual clinical effects of mirror therapy and the related neuro-mechanism behind the reduction of spatial neglect in post-stroke patients. One early study was conducted by Dohle and colleagues [14]. This study examined the effects of using mirror therapy on 36 patients with sub-acute stroke, 20 of whom were suffering from spatial neglect. By using a five-point, self-designed behavioral ratings (based on cancellation, drawing, and bisection tests, as well as omissions and reaction times), they found significant between-group differences in favor of mirror therapy. The mirror visual feedback (MVF) of watching self-induced movements in the neglected hemifield was postulated to be responsible for improvements in spatial neglect [8]. A recent randomized controlled trial of mirror therapy, which was compared to a comparison group using a covered mirror, with 48 patients shows maximum benefits on the star cancellation test at the six-month follow-up, which indicates that MVF benefits patients experiencing spatial neglect [15]. However, because it is uncommon to use a covered mirror in clinical practice, the results of this study were unable to provide conclusive proof that neglect can be improved through MVF of the non-hemiparetic arm. The results of a recent Cochrane review also indicated that no clear effect could be drawn on MVF for improving visuospatial neglect [16]. However, there is evidence to support that both action execution with MVF and action observation training can be effective in activating the parietal-frontal areas in the brain that encompass the mirror neuron system [17]. To address this, we used a transparent glass wall as sham, which also allowed an action observation of the hemiparetic or neglected arm and is similar to the methods of bimanual arm training, and we then compared this with the effects of using a covered mirror. We hypothesized that MVF would be associated with stronger visuomotor integration for spatial neglect deficits than observing the movement of the hemiparetic arm through the glass wall. The objective of this study was to investigate the effects of MVF, with reference to using a transparent glass wall and using a covered mirror, on reductions in spatial neglect for patients with stroke in a preliminary randomized controlled study.

## 2. Materials and Methods

### 2.1. Participants

The participants in this study were adult inpatients with subacute stroke (onset ≤ six months), recruited through convenience sampling from two hospitals in Hong Kong and Guangzhou, during the one and a half years of the study. The choice of a poststroke period of less than six months as an inclusion criterion for stroke reflected patients’ maximal point of recovery during the period, as a past study shows that at six months after stroke, 52% and 46% of patients had recovered completely from personal neglect and neglect of far space, respectively [18]. This study was a single-blinded randomized controlled trial that compared three groups: (a) MVF group, with a mirror illusion applied to the non-affected arm; (b) sham 1 group, in which participants viewed the affected arm through a transparent glass wall; and (c) sham 2 group in which participants’ view of the affected arm was restricted through the use of a covered mirror. The use of a covered mirror for comparison was to control for the transfer effect of non-hemiparetic hand (i.e., the right hand) motor training on the performance of the hemiparetic hand and the attention of the hemifield [19]. Patients were randomly assigned to one of the three groups using block randomization (blocks of four) with a random numbers table. A researcher who was not aware of the study’s aims and was not involved in the measurements nor subsequent interventions carried out the randomization. Blinded measurements were administered at pre, post, and three-week follow-up stages. The inclusion criteria were: (1) ischemic or hemorrhagic stroke, confirmed by medical diagnoses compatible with unilateral right lesion involvement (i.e., left hemiplegic), and exhibited by left visual field inattention, or spatial neglect, by obtaining a total score in the star cancellation subtest of the Behavioral Inattention Test conventional tests (BIT) ≤ 51 (out of 54) [20]; (2) stroke with the onset of a neurological condition ≤ six months previously; (3) normal or corrected-to-normal visual acuity (defined as being greater than 20/60 or 6/18 in the better eye) [21]; (4) hemiplegic upper extremity functional levels between three and seven, as rated by the Functional Test for the Hemiplegic Upper Extremity and able to move against gravity [22]. This test is commonly used for triage of hemiparetic arm severity, and higher scores represent a higher level of hemiparetic arm functioning; (5) the ability to understand and follow simple verbal instructions, with a Mini-Mental State Examination score ≥ 21 [23]; (6) the ability to participate in a therapy session lasting at least 30 min; and (7) able to give informed consent to participant in the study. Individuals who met the following exclusion criteria were not permitted to participate in the study: (1) prior neurological or psychiatric disorders; (2) severe spasticity (Modified Ashworth Scale > 3) over the paretic arm [24]; (3) a history of recent Botox injections or acupuncture to the paretic upper extremity within the past three months; and/or (4) participation in another clinical study elsewhere during the recruitment period. This study was carried out according to the Declaration of Helsinki. Informed written consent was obtained from all patients prior to data collection. The Human Subjects Research Ethics Committee of the Hong Kong Polytechnic University (Ref. no. HSEARS20140305004) and the Institutional Review Board of Hospital Authority West Cluster (Ref. no. UW 15-141) approved the study before enrolment of patients started. The clinical trial registration number of this study (URL: http://www.clinicaltrials.gov accessed on 26 February 2019) is: NCT03854487.

### 2.2. Intervention

**Figure 1:**
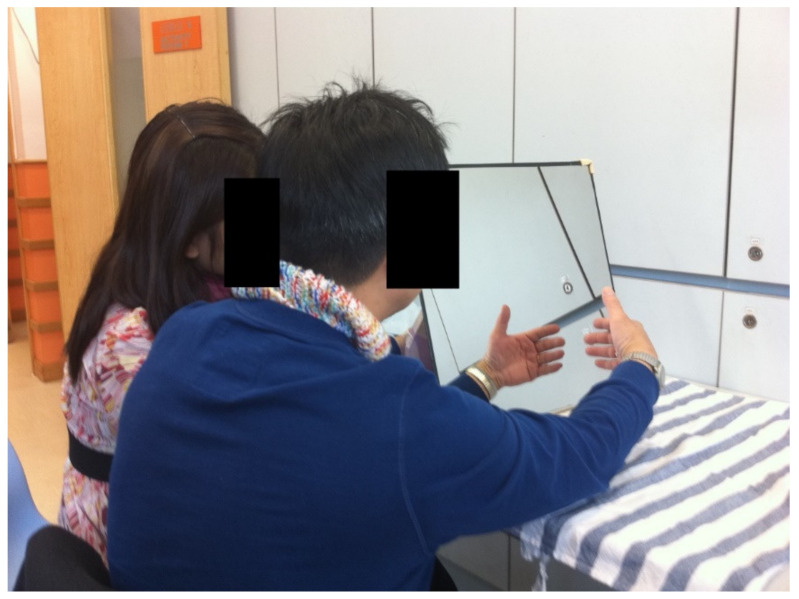
The mirror plane apparatus.

The only piece of equipment used for the training was the mirror plane apparatus, which had dimensions of 16 × 17 inches and was placed at the midsagittal plane of the patient (Figure 1). The treatment program for all groups consisted of 12 sessions (four per week for three weeks), each lasting for 30 min. The length of the 12 sessions of intervention was determined according to the findings of a previous systematic review on the effects of MT on hemiparetic arm after stroke [25]. The movement practice for all groups involved five table-top tasks, lasting for 30 min in total, and it was under the supervision of an occupational therapist (Table 1). The patient was instructed to perform as many trials as possible in each session, with a maximum of 30 trials per task, giving a total of 150 trials per session. Treatment activities (graded according to the severity of the patient’s upper extremity impairment) lasted for 30 min. Patients in all groups performed bimanual upper limb exercises with graded levels of difficulty based on the individual’s level of upper limb function. The exercises were customized and based on the seven functional levels of Functional Test for the Hemiplegic Upper Extremity [22]. The test was developed and validated according to Brunnstorm’s developmental stages of stoke recovery [22]. Each treatment session consisted of five customized table-top tasks, with reference to patients’ functional levels (Table 1) [25]. All patients in this study followed other conventional therapies (psychological, physical therapy, occupational therapy, etc.) in addition to the treatments described in this intervention.

The patients in the MVF group watched the movements of the non-affected arm in the mirror and actively tried to imitate them with the affected limb, synchronizing it with the reflection [12,14,26]. In the sham 1 group, the patient followed the same protocol as in the MVF group but with a direct view of the affected arm through a transparent glass wall. In the sham 2 group, the mirror was covered by a cloth and the patient was instructed to move both arms while looking at a cross mark on the covered mirror, while imagining the analogous movements of the affected arm; this group otherwise followed the same protocol as in the MVF group. The only difference among the groups was the presence of the mirror illusion of the non-affected arm or a direct view of the affected arm through glass wall. If the patient was unable to do the tasks because of the severity of their arm impairments, an occupational therapist provided facilitation to the affected arm in the participants’ active movements, so as to synchronize it with the reflection of the movements of the unaffected arm in the mirror [12,14,26]. In order to make sure the patient was continuously looking at the mirror image, the therapist stood behind the patient, who was asked to turn his or her head and eyes to the left to view the mirror and was asked “What is this?” [27] The moving non-affected arm was not covered or hidden from sight to ensure that the space was large enough to allow a wide range of table-top tasks to be practiced.

### 2.3. Outcome Measurement

Data were collected at three intervals: at baseline, three-week post-treatment, and at a three-week follow-up. One principle assessor, who was unaware of the group membership, was responsible for all measurements taken throughout the duration of the study. However, the occupational therapists who were responsible for carrying out the experimental, sham 1, and sham 2 treatments were aware of the group membership and were also responsible for the intervention, as allocated by the data manager. First, information was collected on the patients’ demographic characteristics, medical histories, and functional levels of the Functional Test for the Hemiplegic Upper Extremity.

The primary outcome measures included assessments of spatial neglect using: (1) the Behavioral Inattention Test conventional tests (BIT) [20], (2) the Gap Detection Test (GDT) [28], and (3) the Catherine Bergego Scale (CBS) [29]. The BIT is the most commonly used test for UN. It is divided into two categories consisting of conventional and behavioral subtests. The conventional subtests comprise six items: line crossing, letter cancellation, star cancellation, figure and shape copying, line bisection, and representational drawing. The behavioral subtests comprise nine items: picture scanning, telephone dialing, menu reading, article reading, map reading, coin sorting, and address and sentence copying. The total score for the conventional subtests is 160, while it is 81 for the behavioral subtests [20]. Both egocentric and allocentric forms of neglect can be differentiated by means of a GDT [28], which consists of two figure discriminating tasks: the circle discriminative cancellation task and the triangle discriminative cancellation task. The stimulus sheet is placed on the desk at the midsagittal plane of the patient’s body. The patient is instructed to circle every complete circle or triangle and to cross out every incomplete circle or triangle with a pen held in the right hand [28]. There are three scores: the percentage of figures omitted (no response to the stimulus), the percentage of left-gap missed, and the percentage of right-gap missed. In this study, egocentric neglect was scored by counting the number of total figures identified on the left (hemi-space) and right sides of the page, while allocentric neglect was scored by counting the number of complete right-gap and left-gap figures identified on the left (hemi space) and right sides of the page. The higher the score, the greater the improvement in regard to neglect experienced by the patient. The CBS is a functional scale consisting of 10 items related to the observation of spatial neglect in regard to peri-personal or extrapersonal space in activities of daily living. The severity of neglect is scored through observations of each item on a four-point scale [29].

The secondary outcome measure was upper extremity motor functions, assessed using the Fugl–Meyer assessment [30,31]. The upper limb subscore of the Fugl–Meyer Assessment is a measure of the synergistic pattern of and ability to make arm movements. It consists of a three-point scale with a total maximum score of 66. The total score can be further divided into arm and hand subscores [30].

### 2.4. Statistical Analysis

Data analysis was conducted using SPSS version 23.0 (SPSS Inc., Madison, WI, USA). Demographic and baseline characteristics were compared using an analysis of variance (ANOVA; continuous and ordinal data) or chi-square tests (categorical data). Intention-to-treat analysis was performed using the “last observation carried forward” (LOCF) method. A mixed-effects model with random intercepts and slopes was used to detect any significant difference in the rate of change in behavioral outcomes among the three groups. Group effects, time effects, and group-by-time interaction effects were included as fixed effects. The random intercept and random slope of change in the dependent variable over time were included as random effects. Any unbalanced demographical and baseline variables were included as covariates in the mixed-effects model. Maximum likelihood estimation was chosen as the estimation method and the covariance structure was assumed to be unstructured. A significant group-by-time interaction effect indicated that the dependent variable changed at different rates between the groups. A significant time effect indicated that the dependent variable changed significantly within a single group. A *p* value of 0.05 or lower was considered to indicate significance.

## 3. Results

**Figure 2:**
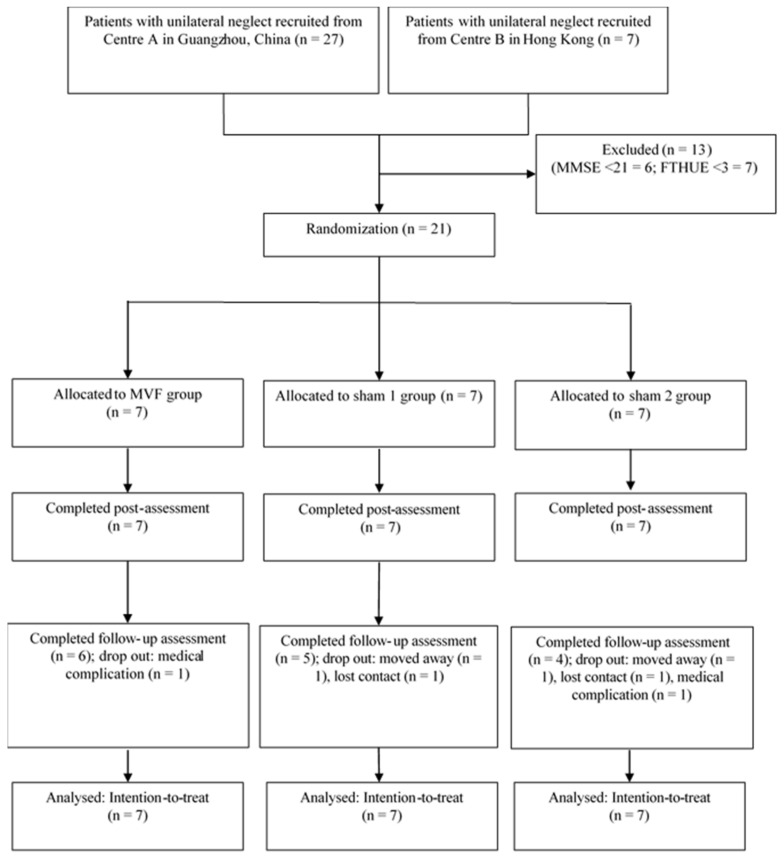
CONSORT flowchart of participant recruitment. MVF = mirror visual feedback; MMSE = Mini-Mental State Examination; FTHUE = Functional Test for the Hemiplegic Upper Extremity.

A total of 21 inpatients (MVF = 7; sham 1 = 7; sham 2 = 7) were included in the present study. Figure 2 depicts a flowchart of the recruitment process. At follow-up assessment, there was one dropout in the MVF group, two dropouts in the sham 1 group, and three dropouts in the sham 2 group. Data from all 21 patients were used in the final analysis, after conducting the LOCF process. There were no significant differences among the three groups in their demographic and baseline characteristics, aside from in regard to the Mini-Mental State Examination (*p* = 0.036) (see Table 2 for details). This variable was therefore included as a covariate in the mixed-effects model.

### 3.1. Primary Outcome: Spatial Neglect

**Figure 3:**
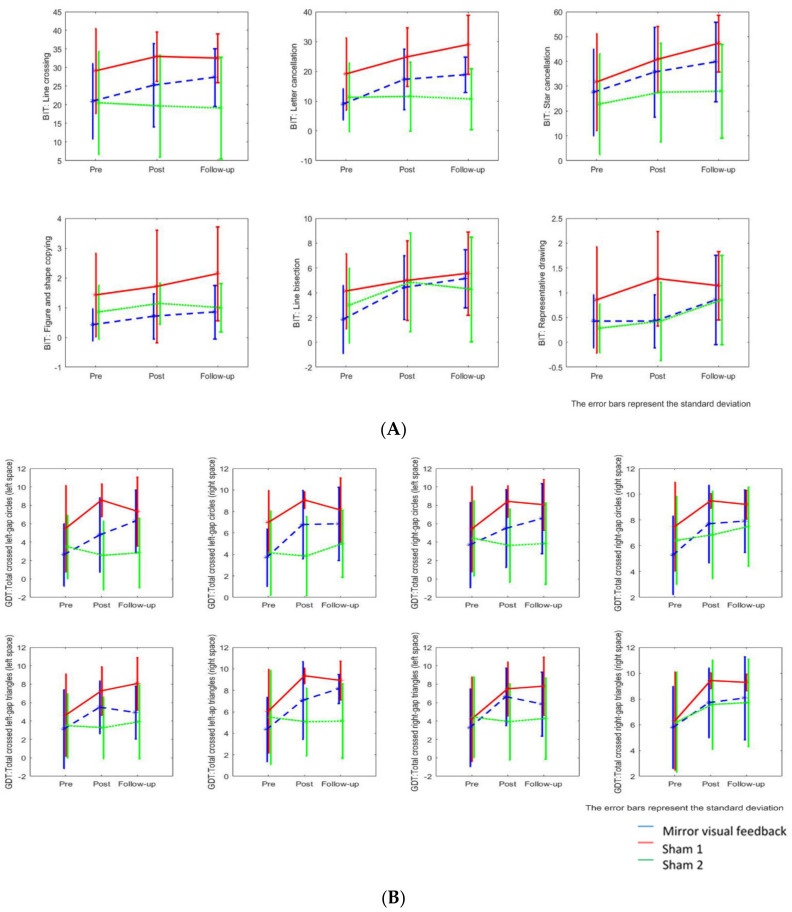
Changes in the Behavioral Inattention Test conventional tests and the Gap Detection Test in the three groups across time. (**A**) Changes in the Behavioral Inattention Test conventional subtests. (**B**) Changes in the Gap Detection Test (crossed circles and triangles).

Figure 3 shows the changes in the BIT & the GDT in the three groups across time. Table 3 shows the results of the behavioral outcomes across the three groups.

In regard to the total score of the BIT, the mixed-effects model demonstrated significant time effects in both the MVF (β = 16.36, *p* = 0.005) and sham 1 (β = 16.00, *p* = 0.005) groups, but not in the sham 2 group (β = 2.57, *p* = 0.623). Significant time effects were found in the letter cancellation, line crossing, and line bisection subtests for both the MVF and sham 1 groups but not in the sham 2 group. A significant group-by-time interaction effect was only observed in the line crossing subtest of the BIT, when comparing participants in the MVF group with those in sham 2 (Δβ = 5.21, *p* = 0.022). An insignificant group-by-time interaction effect, very close to significance, was observed in the letter cancellation subtest of the BIT, when comparing participants in the MVF group with those in sham 2 (Δβ = 3.93, *p* = 0.056).

In regard to the CBS, the mixed-effects model demonstrated significant time effects in all three groups (all *ps* < 0.05). However, no significant group-by-time interaction effect was noted.

Significant time effects were noted in both the MVF and sham 1 groups in most subtests of the GDT (circle discrimination). For the subtests that required participants to discriminate the incomplete circles (left-gap) in either the left or right space, a significant time effect was only noted in the MT group (*ps* = 0.003), not in the sham 1 or sham 2 groups. However, a significant group-by-time interaction effect was only observed in the subtest in which participants were required to discriminate the incomplete circles (left-gap) at their left space, when comparing participants in the MT group with those in sham 2 (Δβ = 2.14, *p* = 0.013). Significant time effects were noted in both the MVF and sham 1 groups in most subtests of the GDT (triangle discrimination). A significant group-by-time interaction effect was only observed in the test in which participants were required to discriminate the incomplete triangles (left-gap) on their right space, when comparing participants in the MVF group with those in the sham 2 group (Δβ = 2.07, *p* = 0.010).

### 3.2. Secondary Outcome: Upper Limb Motor Functions

Significant time effects were only observed in the MVF group, not in the sham 1 or sham 2 groups, in the total score of the Fugl–Meyer Assessment (β = 5.21, *p* = 0.017) and its arm subscore (β = 3.21, *p* = 0.013). No significant time effect was found in the hand subscore of the Fugl–Meyer Assessment, aside from a marginally time effect found in the MVF group (β = 2.00, *p* = 0.067). No significant group-by-time interaction effect was noted in the Fugl–Meyer Assessment or its subscores.

## 4. Discussion

Our findings confirm that both MVF is better than sham 2 (using a covered mirror) in reducing spatial neglect. These results, indicating that MVF is superior to therapy using a covered mirror, are consistent with the results of another randomized controlled study [15]. However, in the present study, when MVF is compared with sham 1 (in which the patient can see the affected arm through a transparent glass wall and which is similar to bimanual arm training with both arms simultaneously moving together), participants in both groups demonstrate improvements in spatial neglect, with those in the MVF group performing slightly better than those in sham 1 in certain cancellation and discrimination tasks. As it is uncommon to use a covered mirror in clinical practice, this additional finding is useful in informing our understanding of the findings of Pandian and colleagues, who investigated whether or not MVF from observation of the non-hemiparetic arm is an effective method for reducing spatial neglect [15]. This finding is also consistent with the findings of our recent published review on the effects of action observation and MVF on neuroplasticity in stroke that both MVF and action observation (seeing the affected arm during bimanual movement) may contribute to stroke recovery by revising the interhemispheric imbalance caused by stroke due to the activation of the MNS [17]. However, MVF gives incongruent visual feedback induced by the mirror, which makes it different from bimanual arm training. A recent electroencephalography (EEG) study supports the view that MVF can decrease hemispheric asymmetry and re-establish the hemispheric balance that has been disrupted by stroke [32]. Therefore, in the current study, it is likely that the mirror mediates the participants’ recognition of the mirror illusion before and after MVF, compared to participants in the sham 1 and sham 2 groups, evidencing some advantages of the cancellation tasks.

Overall, MVF demonstrated no significant advantage for reducing spatial neglect over sham 2—the alternative method of viewing the hemiparetic arm through a transparent glass wall during bilateral arm movement. However, it is interesting to note that MVF was slightly more beneficial than sham 2 in terms of aiding participants to discriminate between the left-gap circles at the left space and the left-gap at the right space but not in regard to the total number of circles or triangles identified on the left and right sides of the page in the GDT. This finding has important implications about the heterogeneity of neglect, which shows that the mirror illusion in MVF is slightly more effective in alleviating allocentric symptoms, which are detected by the omission of or inattention to the contralesional side of a stimulus, regardless its location in relation to the body of the viewer. As UN is strongly associated with the disruption of inter-hemispheric connectivity in the dorsal attention network [4], this finding led to the present paper’s formulation of a hypothesis that visual mirror feedback, similar to the findings in our previous study, contributes to recovery from neglect by recruiting the parieto-frontal mirror circuit and rebalancing the interhemispheric activities of the left and right peri-personal or extrapersonal spaces. This encompasses the mirror neurons system in terms of decreasing the asymmetry of event-related desynchronization in alpha frequency range [33]. However, our post-stroke screening did not allow for a clear distinction to be made in regard to symptoms between the egocentric and allocentric subtypes of neglect during the recruitment of the patients. This situation echoes that of another recent study on the use of a cancellation task and an observational assessment, which might not be sensitive enough to detect neglect and its heterogeneity [34].

In line with the findings of our previous study on spatial neglect, we found that the observed improvements in regard to neglect could not be generalized into gains in functional independence or motor recovery [35]. No significant results were observed at the functional level when measured using the CBS. However, we found a slight advantage of using MVF over sham 1 and sham 2 in improving the arm functioning measured using the FMA. Although most of our patients with spatial neglect suffered from severe arm impairments and lower functioning, rated levels 1-4 in the FTHUE, mirror therapy brings benefits precisely in the subacute phase when the patient has no muscle strength and by stimulating the mirror neurons, in parallel with an associated decrease in spatial neglect.

Our study has some limitations. It was quite rigorous in that it was a single-blind randomized trial conducted at two trial sites with three groups, including two sham therapy groups. This created the difficulty of needing to recruit more patients in order to reach the correct estimated power. In addition, a subgroup analysis on the arm functioning based on the upper extremity functional levels was impossible due to the small sample. The sample size was smaller in each group than we had predicted. All participants in our study had right hemispheric stroke, therefore, the results could only be used for the population of patients with spatial neglect following right hemispheric stroke. Moreover, the process of participant recruitment in this study lasted one and a half years, much longer than expected, because most of the patients were severely hemiparetic. The dropout rate was also high; many resignations from the study were due to instability in medical status, being referred back to acute medical management, or patients were unable to attend follow-up appointments because of their admission to residential institutions or infirmaries after inpatient discharge. To account for this, we used mixed-effects models in order to accommodate the subjects with missing values. In addition, the neglect improved in the sham 2 group because of the spontaneous recovery at the subacute stage by the time of the study [36], thus, it was more difficult for the MVF group to reach a statistically significant result, compared to the sham 2 group. The fact that the therapists assisted participants with movements of the affected arm to synchronize them to the unaffected arm might be seen as constituting a potential confounding effect, arising from this additional tactile cue from the therapist’s hand on the participant’s skin, hence, leading to unequal tactile cues between the affected and unaffected limbs. Finally, the intensity of the 12-sessions of therapy might be too small to provide relevant effects for patients with spatial neglect. In future, the number of sessions should be extended to more than 12 sessions in 3 weeks for more promising results.

## 5. Conclusions

Our study confirms that MVF was superior to using a covered mirror as a method for reducing spatial neglect and for alleviating its allocentric symptoms, and it was in parallel with an improvement in arm functioning of the hemiplegic upper extremity. However, MVF demonstrated no significant advantage in improving neglect over the alternative method of viewing the hemiparetic arm through a transparent glass wall during bilateral arm movement.

## Figures and Tables

**Table 1:** Table-top tasks for task-specific training during mirror visual feedback.

Corresponding Levels in FTHUE ^1^	Principles of Movement	Tasks Recommended ^#^
1–3	1. Range of motion exercise 2. Limb segments working together as functional synergy	1. Elbow flexion and extension with hand in resting position 2. Forearm pronation with full fist
3–4	1. Range of motion exercise 2. Limb segments working together as functional synergy	1. Wrist flexion and extension with elbow support on table 2. Elbow flexion and extension with finger extension
4–5	1. Individual limb segments control training 2. Grasp and release training	1. Grasp and release (with cylindrical grasp) 2. Wrist circumduction with fingers in prayer position
5–6	1. Individual finger movement 2. Grasp and release training	1. Finger opposition 2. Grasp and release (with soft ball)
6–7	1. Fine motor skills training 2. Endurance, speed, and coordination in arm use	1. Pen shifting using fingers 2. Card translation between fingers

^1^ FTHUE = Functional Test for the Hemiplegic Upper Extremity; # repeated at least 10 times for each item.

**Table 2:** Comparison of demographic and baseline characteristics.

Variable	MVF	Sham 1	Sham 2	Between-Group Comparisons (*p)*
	(n = 7)	(n = 7)	(n = 7)	
Age (years) (mean, SD)	63.86 (8.78)	49.57 (12.46)	61.86 (14.90)	0.09
Gender: Female (n, %)	3 (42.9%)	1 (14.3%)	1 (14.3%)	0.35
Time after onset (days) (mean, SD)	34.57 (35.68)	89.29 (106.77)	76.29 (54.14)	0.354
Type				
Ischemic (n, %)	5 (71.4%)	4 (57.1%)	1 (14.3%)	0.084
Hemorrhagic (n, %)	2 (28.6%)	3 (42.9%)	6 (85.7%)	
MMSE (mean, SD)	19.00 (5.80)	25.14 (4.18)	20.00 (2.38)	0.036 *
FTHUE levels (n, %)				
1	5 (71.4)	3 (42.9)	5 (71.4)	0.675
2	1 (14.3)	2 (28.6)	2 (28.6)	
3	0 (0)	1 (14.3)	0 (0)	
4	1 (14.3)	1 (14.3)	0 (0)	

* *p* ≤ 0.05; values were n (%) or mean (SD). MVF = mirror visual feedback; FTHUE = Functional Test for the Hemiplegic Upper Extremity; FMA = Fugl–Meyer Assessment upper extremity subscore; MMSE = Mini-Mental State Examination; BIT = Behavioral Inattention Test conventional tests; CBS = Catherine Bergego Scale.

**Table 3:** Results of the behavioral outcomes across the three groups.

			Descriptive Means (SD)			Time Effects	Group-by-Time Interaction Effects			
			Pre	Post	FU	*p*	Comparisons	Δβ	95% CI	*p*
BIT	Conventional total score	MVF	60.29 (30.48)	83.86 (35.15)	93.00 (23.81)	0.005 **	1, 2	0.36	−14.78–15.50	0.961
		Sham 1	86.57 (43.70)	106.71 (30.61)	118.57 (28.27)	0.005 **	1, 3	13.79	−1.35–28.93	0.072
		Sham 2	58.86 (43.73)	65.29 (37.41)	64.00 (37.24)	0.623				
	Line crossing	MVF	21.00 (10.13)	25.29 (11.22)	27.43 (7.74)	0.003 **	1, 2	0.00	−4.40–4.40	0.999
		Sham 1	29.14 (11.41)	33.00 (6.63)	32.57 (6.53)	0.003 **	1, 3	5.21	0.81–9.62	0.022 *
		Sham 2	20.57 (13.77)	19.71 (13.71)	19.14 (13.69)	0.850				
	Letter cancellation	MVF	9.00 (5.20)	17.29 (10.14)	18.86 (5.93)	0.029 *	1, 2	1.50	−2.54–5.54	0.449
		Sham 1	19.14 (12.13)	24.86 (9.91)	29.00 (9.97)	0.226	1, 3	3.93	−0.11–7.97	0.056
		Sham 2	11.29 (11.49)	11.57 (11.57)	10.71 (10.27)	−0.609				
	Star cancellation	MVF	27.57 (17.52)	35.71 (18.18)	39.86 (15.99)	0.061	1, 2	−1.64	−10.77–7.48	0.712
		Sham 1	31.71 (19.53)	40.86 (13.25)	47.29 (11.46)	0.020 *	1, 3	3.57	5.55–12.70	0.425
		Sham 2	22.85 (20.21)	27.57 (19.84)	28.00 (18.84)	0.417				
	Figure and shape copying	MVF	0.43 (0.53)	0.71 (0.76)	0.86 (0.90)	0.354	1, 2	−0.14	−0.81–0.52	0.659
		Sham 1	1.43 (1.40)	1.71 (1.89)	2.14 (1.57)	0.129	1, 3	0.14	−0.52–0.81	0.659
		Sham 2	0.86 (0.90)	1.14 (0.69)	1.00 (0.82)	0.775				
	Line bisection	MVF	1.86 (2.73)	4.43 (2.57)	5.14 (2.34)	0.033 *	1, 2	0.93	−1.20–3.05	0.373
		Sham 1	4.14 (3.02)	5.00 (3.21)	5.57 (3.36)	0.334	1, 3	1.00	−1.12–3.12	0.339
		Sham 2	3.00 (3.00)	4.86 (3.98)	4.29 (4.23)	0.383				
	Representative drawing	MVF	0.43 (0.53)	0.43 (0.53)	0.86 (0.90)	0.220	1, 2	0.07	−0.43–0.57	0.768
		Sham 1	0.86 (1.07)	1.29 (0.95)	1.14 (0.69)	0.408	1, 3	−0.07	−0.57–0.43	0.768
		Sham 2	0.29 (0.49)	0.43 (0.79)	0.86 (0.90)	0.106				
CBS	Total score	MVF	13.77 (6.68)	8.23 (7.58)	8.58 (8.62)	0.026 *	1, 2	1.86	−1.34–5.06	0.241
		Sham 1	15.17 (9.00)	10.59 (5.06)	6.26 (5.20)	0.001 **	1, 3	0.05	−3.15–3.25	0.974
		Sham 2	15.68 (9.45)	11.78 (6.06)	10.39 (5.60)	0.024 *				
GDT	Total crossed circle (left space)	MVF	11.50 (14.59)	16.86 (13.80)	20.50 (10.95)	0.023 *	1, 2	−0.07	−5.47–5.33	0.978
		Sham 1	16.43 (14.11)	25.93 (5.18)	25.57 (5.67)	0.021 *	1, 3	4.61	−0.79–10.01	0.091
		Sham 2	12.36 (11.95)	12.50 (12.87)	12.14 (13.75)	0.954				
	Total crossed circle (right space)	MVF	21.36 (7.94)	24.79 (8.56)	26.14 (6.69)	0.053	1, 2	0.07	−3.37–3.51	0.966
		Sham 1	23.71 (9.23)	29.36 (0.24)	28.36 (2.29)	0.060	1, 3	−0.04	−3.47–3.40	0.983
		Sham 2	19.71 (10.43)	23.00 (9.63)	24.57 (9.03)	0.050 *				
	Total crossed left−gap circle (left space)	MVF	2.64 (3.42)	4.79 (4.05)	6.29 (3.41)	0.003 **	1, 2	0.89	−0.77–2.55	0.284
		Sham 1	5.50 (4.73)	8.57 (1.77)	7.36 (3.74)	0.118	1, 3	2.14	0.48–3.80	0.013 *
		Sham 2	3.50 (3.49)	2.57 (3.75)	2.86 (3.77)	−0.583				
	Total crossed left−gap circle (right space)	MVF	3.71 (2.69)	6.79 (3.17)	6.86 (3.40)	0.003 **	1, 2	1.00	−0.45–2.45	0.173
		Sham 1	7.00 (2.99)	9.07 (0.79)	8.14 (3.01)	0.270	1, 3	1.14	−0.30–2.59	0.120
		Sham 2	4.14 (3.97)	3.86 (3.67)	5.00 (3.12)	0.407				
	Total crossed right−gap circle (left	MVF	3.71 (4.67)	5.50 (4.23)	6.57 (3.82)	0.024 *	1, 2	0.11	−1.62–1.83	0.898
		Sham 1	5.43 (4.64)	8.43 (1.72)	8.07 (2.75)	0.035 *	1, 3	1.71	−0.01–3.44	0.051
	space)	Sham 2	4.43 (4.13)	3.64 (3.99)	3.86 (4.40)	0.631				
	Total crossed right−gap circle (right space)	MVF	5.29 (3.05)	7.71 (3.01)	7.93 (2.42)	0.006 **	1, 2	0.46	−0.81–1.74	0.456
		Sham 1	7.50 (3.46)	9.50 (0.58)	9.21 (1.11)	0.061	1, 3	0.79	−0.49–2.06	0.213
		Sham 2	6.43 (3.43)	6.86 (3.40)	7.50 (3.07)	0.229				
	Total crossed triangle (left space)	MVF	11.36 (14.40)	20.21 (10.40)	19.07 (10.44)	0.038 *	1, 2	−1.32	−6.46–3.81	0.598
		Sham 1	14.36 (13.73)	23.00 (8.18)	24.71 (8.69)	0.007 **	1, 3	3.04	−2.10–8.17	0.233
		Sham 2	12.43 (13.07)	12.71 (12.59)	14.07 (13.66)	0.643				
	Total crossed triangle (right space)	MVF	18.57 (9.41)	25.36 (7.86)	28.21 (2.16)	0.003 **	1, 2	1.21	−2.93–5.36	0.549
		Sham 1	21.00 (9.28)	29.21 (0.95)	28.21 (2.90)	0.018 *	1, 3	2.93	−1.21–7.07	0.156
		Sham 2	19.64 (11.47)	23.14 (9.28)	23.43 (9.57)	0.193				
	Total crossed left−gap triangle (left space)	MVF	3.14 (4.30)	5.50 (2.87)	4.93 (2.86)	0.162	1, 2	−0.82	−2.63–0.99	0.356
		Sham 1	4.64 (4.51)	7.29 (2.63)	8.07 (2.85)	0.011 *	1, 3	0.68	−1.13–2.49	0.444
		Sham 2	3.50 (3.52)	3.29 (3.34)	3.93 (3.98)	0.731				
	Total crossed left−gap triangle (right space)	MVF	4.36 (3.02)	7.07 (3.61)	8.14 (1.35)	0.001 **	1, 2	0.46	−1.08–2.01	0.547
		Sham 1	6.07 (3.89)	9.36 (0.75)	8.93 (1.79)	0.012 *	1, 3	2.07	−0.52–3.62	0.010*
		Sham 2	5.50 (4.40)	5.07 (3.14)	5.14 (3.47)	0.743				
	Total crossed right−gap triangle (left space)	MVF	3.29 (4.24)	6.64 (3.13)	5.86 (3.47)	0.030 *	1, 2	−0.50	−2.13–1.13	0.529
		Sham 1	4.21 (4.60)	7.50 (2.94)	7.79 (3.15)	0.004 **	1, 3	1.36	−0.27–2.98	0.097
		Sham 2	4.43 (4.42)	3.93 (4.13)	4.29 (4.39)	0.898				
	Total crossed right−gap triangle (right space)	MVF	5.79 (3.20)	7.71 (2.71)	8.07 (3.22)	0.047 *	1, 2	−0.36	−1.95–1.24	0.646
		Sham 1	6.29 (3.83)	9.43 (0.61)	9.29 (0.64)	0.011 *	1, 3	0.40	−2.00–1.20	0.613
		Sham 2	6.21 (3.91)	7.57 (3.47)	7.71 (3.39)	0.181				
FMA	Total score	MVF	8.71 (10.00)	16.43 (18.41)	19.14 (20.34)	0.017 *	1, 2	1.57	−4.37–7.51	0.588
		Sham 1	11.71 (13.43)	15.14 (17.07)	19.00 (22.00)	0.085	1, 3	4.29	−1.65–10.22	0.148
		Sham 2	9.14 (8.34)	10.00 (8.19)	11.00 (8.72)	0.650				
	Upper extremity subscore	MVF	7.57 (8.58)	11.85 (10.79)	14.00 (12.19)	0.013 *	1, 2	1.29	−2.20–4.78	0.452
		Sham 1	9.00 (9.18)	11.29 (10.58)	12.86 (11.61)	0.119	1, 3	2.57	−0.92–6.06	0.140
		Sham 2	7.43 (4.89)	8.14 (4.67)	8.71 (5.00)	0.594				
	Hand subscore	MVF	1.14 (1.95)	4.57 (7.81)	5.14 (8.86)	0.067	1, 2	0.29	−2.76–3.33	0.847
		Sham 1	2.71 (4.35)	3.86 (6.64)	6.14 (10.49)	0.112	1, 3	1.71	−1.33–4.76	0.254
		Sham 2	1.71 (3.73)	1.86 (3.76)	2.29 (4.07)	0.785				

* p ≤ 0.05; ** *p* ≤ 0.01. SD: standard deviation; Δβ: difference in slope; CI: confidence interval; MVF = mirror visual feedback; BIT = Behavioral Inattention Test conventional tests; CBS = Catherine Bergego Scale; GDT = Gap Detection Test; FMA = Fugl–Meyer Assessment.

## Data Availability

Not applicable.
